# *Escherichia coli* as a New Platform for the Fast Production of Vault-like Nanoparticles: An Optimized Protocol

**DOI:** 10.3390/ijms232415543

**Published:** 2022-12-08

**Authors:** Roger Fernández, Aida Carreño, Rosa Mendoza, Antoni Benito, Neus Ferrer-Miralles, María Virtudes Céspedes, José Luis Corchero

**Affiliations:** 1 Institut de Biotecnologia i de Biomedicina, Universitat Autònoma de Barcelona, Bellaterra, 08193 Barcelona, Spain; 2 CIBER de Bioingeniería, Biomateriales y Nanomedicina, Instituto de Salud Carlos III, Bellaterra, 08193 Barcelona, Spain; 3 Laboratori d’Enginyeria de Proteïnes, Departament de Biologia, Universitat de Girona, 17003 Girona, Spain; 4 Institut d’Investigació Biomèdica de Girona Josep Trueta, (IdIBGi), 17190 Salt, Spain; 5 Departament de Genètica i de Microbiologia, Universitat Autònoma de Barcelona, Bellaterra, 08193 Barcelona, Spain; 6 Grup d’Oncologia Ginecològica i Peritoneal, Institut d’Investigacions Biomédiques Sant Pau, Hospital de Santa Creu i Sant Pau, 08041 Barcelona, Spain

**Keywords:** prokaryotic cell factories, vault particles, protein nanocages, drug-delivery systems, self-assembling protein nanoparticles

## Abstract

Vaults are protein nanoparticles that are found in almost all eukaryotic cells but are absent in prokaryotic ones. Due to their properties (nanometric size, biodegradability, biocompatibility, and lack of immunogenicity), vaults show enormous potential as a bio-inspired, self-assembled drug-delivery system (DDS). Vault architecture is directed by self-assembly of the “major vault protein” (MVP), the main component of this nanoparticle. Recombinant expression (in different eukaryotic systems) of the MVP resulted in the formation of nanoparticles that were indistinguishable from native vaults. Nowadays, recombinant vaults for different applications are routinely produced in insect cells and purified by successive ultracentrifugations, which are both tedious and time-consuming strategies. To offer cost-efficient and faster protocols for nanoparticle production, we propose the production of vault-like nanoparticles in *Escherichia coli* cells, which are still one of the most widely used prokaryotic cell factories for recombinant protein production. The strategy proposed allowed for the spontaneous encapsulation of the engineered cargo protein within the self-assembled vault-like nanoparticles by simply mixing the clarified lysates of the producing cells. Combined with well-established affinity chromatography purification methods, our approach contains faster, cost-efficient procedures for biofabrication in a well-known microbial cell factory and the purification of “ready-to-use” loaded protein nanoparticles, thereby opening the way to faster and easier engineering and production of vault-based DDSs.

## 1. Introduction

Most therapeutic proteins are administered in their native form and, subsequently, face issues that compromise their efficiency such as biodegradation, low membrane permeability, or rapid clearance from the bloodstream. One well-established strategy to minimize such problems is to protect these proteins by combining them with drug-delivery systems (DDSs) [1,2]. The use of synthetic DDSs (such as inorganic nanoparticles) involves unresolved issues such as reproducibility in the synthesis processes, and their therapeutic use can be hampered by toxicity issues or low delivery efficiencies [3]. On the contrary, bio-inspired nanocarriers are promising alternatives because they better satisfy requisites such as the safety profiles, biocompatibility, biodegradability, solubility and non-immunogenicity; they also present functional groups that allow their engineering to improve their performance [4,5]. Examples of nature-derived nanocarriers include protein nanocages such as viruses, ferritin cages, bacterial microcompartments, and many others formed via the self-assembly of protein subunits, which results in cage-like structures [6,7]. Such protein-based nanoparticles (NPs) are composed of multiple copies of one or more types of monomeric protein building blocks that spontaneously self-assemble into highly organized hollow structures that are 10–100 nm in diameter [8]. They possess at least one internal cavity that can serve as a container for different cargos such as enzymes or genetic material, as in viral capsids [9]. In addition, their three different interfaces (internal, external, and inter-subunit) can be engineered to gain new functionalities [10]. Protein NPs show other intriguing attributes that make them highly attractive as biological nanomaterials, such as biocompatibility [11], availability in many cases of crystal structures, and genetic and molecular information, which allow for rational chemical and/or genetic engineering [12,13] and highly repetitive structures that allow for the incorporation and display of multiple copies of new moieties [14]. All of these advantages of NPs have led to their use in numerous applications [15]. Among such natural NPs, eukaryotic vaults have arisen in recent years as a very attractive nanocarrier for diverse types of molecules [16].

Vaults are cytoplasmic NPs (~40 × 70 nm, 13 MDa) that are found in many eukaryotic cells but are absent in prokaryotic ones. They show a barrel-shaped architecture that results from the self-assembly of several copies of three proteins [17,18] with the “major vault protein” (MVP; 96 kDa) as the main vault component (up to 75% of the mass). The production of recombinant MVP in insect cells confirmed that this protein encoded all of the information needed to form the vault [19]. One valuable characteristic of vaults is their ability to encapsulate cargo proteins by fusing them either to the MVP protein’s N-terminus or to the INT peptide (a vault-targeting domain from the vault-interacting VPARP protein) [20]. In solution, the vault is a dynamic structure that shows transient open and closed states; this is called “breathing” [21], a mechanism that allows for the encapsulation of vault-interacting proteins. Adding specific tags at the C-terminus of the MVP monomer results in the exposure of such targeting domains on the vault surface and their attachment to target cells [22], which allows them to target vaults to specific cells, organs, or tissues. Once in contact with their targets, vaults can be internalized in vitro via macropinocytosis or phagocytosis [23]. When considering all of these properties, vaults have become one of the most promising nanovehicles in smart DDSs; they have already been successfully explored for vaccination [24,25,26], protein delivery [20,27], targeting of cell-surface receptors [22], and delivery of therapeutics for lung cancer [25] or glioblastoma [28]. Recombinant vaults are routinely produced in *Spodoptera frugiperda* (*Sf*9) insect cells [19] in which MVP expression directs the self-assembly of vault-like particles on polyribosomes [29]. However, the complexity and cost of this approach have propelled the pursuit of better, alternative cell factories for vault production, such as insect larvae [30] or the yeast *Pichia pastoris* [31]. These expression systems also rendered NPs indistinguishable from native vaults, but they have not reached widespread popularity due to issues such as a low familiarity, restricted access to insect larvae culture or a putative undesired immune response due to non-native post-translational modifications [32].

Bacteria are excellent cell factories due to their simplicity both biologically (in terms of biochemistry and physiology) and from a process perspective [33]. Bacterial processes are cheaper than those that use eukaryotic cells due to lower media costs and shorter times. Among the microorganisms used for recombinant protein production, *Escherichia coli* is the most popular expression system [34]. Its success relies on easy culture methodologies, reduced costs, high yields, and the plethora of molecular biology tools available for engineering its DNA sequences to generate novel functionality. In addition, *E. coli,* which was among the first organisms to have its entire genome sequenced [35], is considered the main workhorse in producing non-glycosylated recombinant proteins in the biopharmaceutical industry; this is reflected by the fact that approximately one-third of all biopharmaceuticals that were approved between 2010 and 2014 were produced using this prokaryotic system [33,36]. To offer new, faster, and cost-effective upstream and downstream strategies for vault-like NP production and purification, we describe in this work the production of vault-like NPs in *E. coli* cells, the easy and fast loading of such NPs with a reporter cargo protein, and the IMAC-based purification of the final loaded NPs.

## 2. Results

### 2.1. MVP-H6 Protein Expression and Solubility

**Figure 1:**
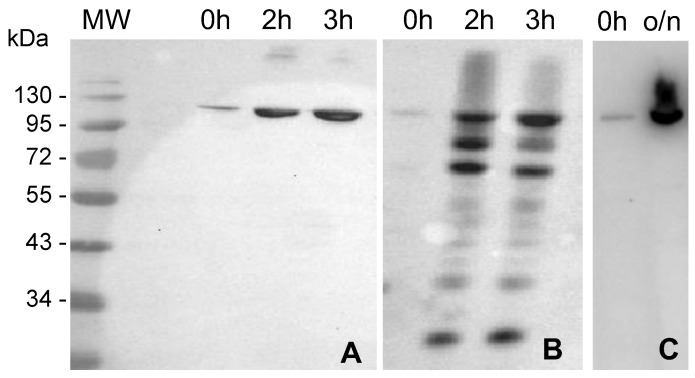
Production of MVP-H6 protein. Total cell pellets obtained from cultures induced either for 3 h at 37 °C (**A**,**B**) or o/n at 20 °C (**C**) were submitted to SDS-PAGE and further Western blots developed with an anti-His monoclonal antibody (**A**,**C**), or with an anti-MVP polyclonal antibody (**B**).

The recombinant protein MVP-H6 was expressed in the BL21(DE3)pLysS strain transformed with pTriEx1.1-MVP-H6 vector. To determine the optimal expression conditions (in terms of protein yield and solubility), the gene expression was induced with IPTG, and the cultures were incubated for either 3 h at 37 °C or overnight (o/n) at 20 °C. MVP-H6 was expressed under both conditions, rendering a product of the expected molecular mass (approx. 100 kDa) that accumulates intracellularly after IPTG addition. Moreover, the recombinant protein was recognized by antibodies against MVP or against His-tag, thus confirming the identity of the recombinant MVP-H6 product. Figure 1 shows the detection of MVP-H6 produced under both conditions.

**Figure 2:**
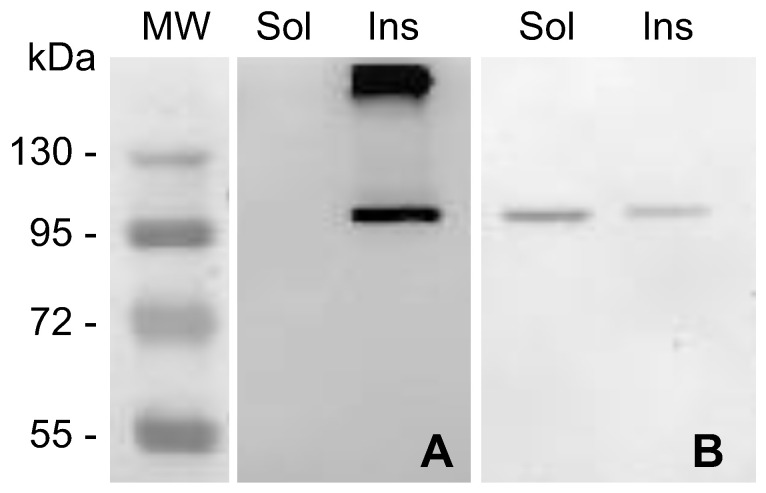
Solubility of MVP-H6 produced in cultures of *E. coli* cells induced with 1 mM IPTG, either for 3 h at 37 °C (**A**) or o/n at 20 °C (**B**). After obtaining soluble (Sol) and insoluble (Ins) cell fractions, they were submitted to SDS-PAGE and further Western blot, developed with an anti-His monoclonal antibody.

The extent of solubility of any recombinant protein is a key factor for its putative application. Therefore, the solubility of the recombinant MVP-H6 was determined. For that, soluble and insoluble fractions from producing cells were initially analyzed by SDS-PAGE and further Western blot. The results (Figure 2) showed that MVP-H6 is mainly found in its insoluble form when produced at 37 °C (Figure 2A). On the contrary, when produced o/n at 20 °C, 66.2 ± 2.9% of the protein remained within the soluble fraction (Figure 2B). According to these results, the induction of gene expression o/n at 20 °C was chosen to produce recombinant MVP-H6 for further experiments. 

**Figure 3:**
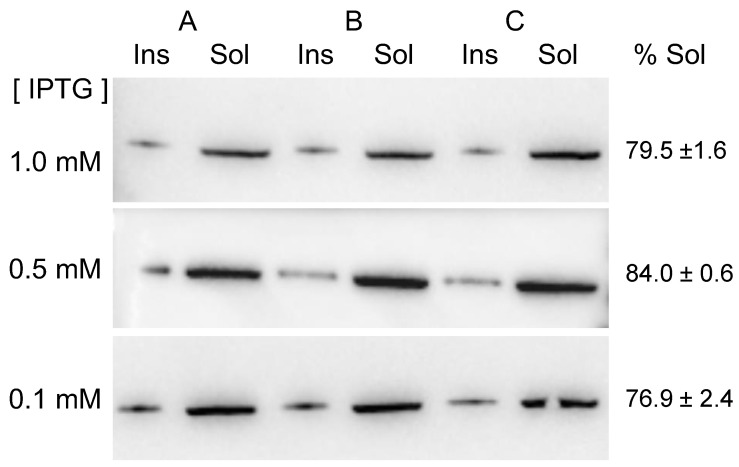
Solubility of MVP-H6 produced at different IPTG concentrations. Three independent cultures (**A**–**C**) were analyzed for each condition. After obtaining insoluble (Ins) and soluble (Sol) cell fractions, they were submitted to SDS-PAGE and further Western blot, developed with an anti-His monoclonal antibody.

To check if protein solubility could be further optimized, induction experiments were repeated but, in this case, including 0.5 and 0.1 mM IPTG as inducer concentrations. The results (Figure 3) showed that in all the tested conditions, the protein was mainly found in its soluble form. Densitometric analyses of the membranes allowed us to estimate that percentages of soluble MVP-H6 were 79.5 ± 1.6, 84.0 ± 0.6 and 76.9 ± 2.4 for 1, 0.5 and 0.1 mM IPTG, respectively.

By the densitometric analyses of membranes containing known amounts of commercial MVP protein, we estimated the amounts of soluble MVP-H6 produced under the different induction conditions, being 8.7 ± 0.7, 28.4 ± 2.7 and 24.0 ± 6.0 mg/L for 1, 0.5 and 0.1 mM IPTG, respectively. In conclusion, we could determine that inducing gene expression in mid-log phase cultures, at 0.5 mM IPTG, with further culture overnight at 20 °C, was the optimal condition for MVP-H6 production.

### 2.2. MVP-H6 Protein Purification

**Figure 4:**
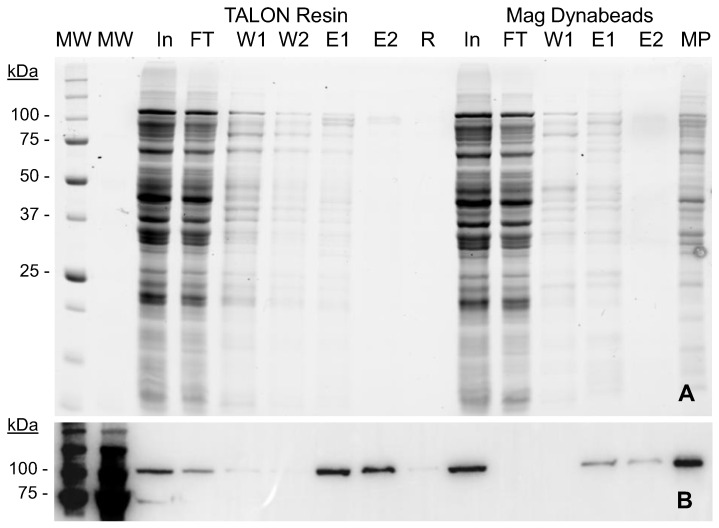
Comparison of TALON resin and magnetic Dynabeads for MVP-H6 purification. Samples were analyzed by (**A**) SDS-PAGE and TGX visualization, and (**B**) Western blot developed with a monoclonal antibody against His-tag. Legend: initial sample (In), flowthrough (FT), first wash (W1), second wash (W2), elution with 250 mM imidazol (E1) and with 2 M imidazol (E2). R and MP correspond, respectively, to samples of resin and magnetic particles after elution steps, in order to evaluate amounts of protein not eluted and still attached to the purification support. MW: molecular weight standard.

The particulate nature of vaults hampers the use of standard packed chromatography columns. In this context, we recently described a protocol to purify vault NPs by means of magnetic separation techniques combined with IMAC chemistries, which allowed us to obtain small amounts of highly pure recombinant vaults [37]. In order to optimize the process, we have compared in this work the protein yield and purity obtained with both magnetic particles and a cobalt-based IMAC resin. The obtained results (Figure 4) showed that MVP-H6 could be captured and further eluted to a significant degree of purity using both platforms. This fact indicates that His-tag is correctly expressed, exposed to the solvent and able to interact with cobalt ions.

However, a deeper analysis revealed clear differences in the performance of the resin compared to the magnetic beads. When using the magnetic support, not all the initially captured protein is later eluted after imidazole treatment. The densitometric analysis of the Western blot (Figure 4B) indicated that less than 50% of the initially captured protein could be later eluted and recovered. This fact suggests that a significant amount of protein remains strongly attached to MPs and cannot be eluted, even at high imidazole concentrations. This assumption was confirmed by boiling and loading the magnetic particles in SDS-PAGE gels (Figure 4B, lane MP), since this process can release the rest of MVP-H6 initially captured. On the contrary, this fact was not observed with the TALON resin. In that case, the attached protein could be easily eluted, and only trace amounts of MVP-H6 were detected when running the resin itself as a sample (Figure 4B, lane R). According to this fact, together with the low binding capacity of magnetic particles previously described, the TALON resin was chosen for further optimization of the process.

**Figure 5:**
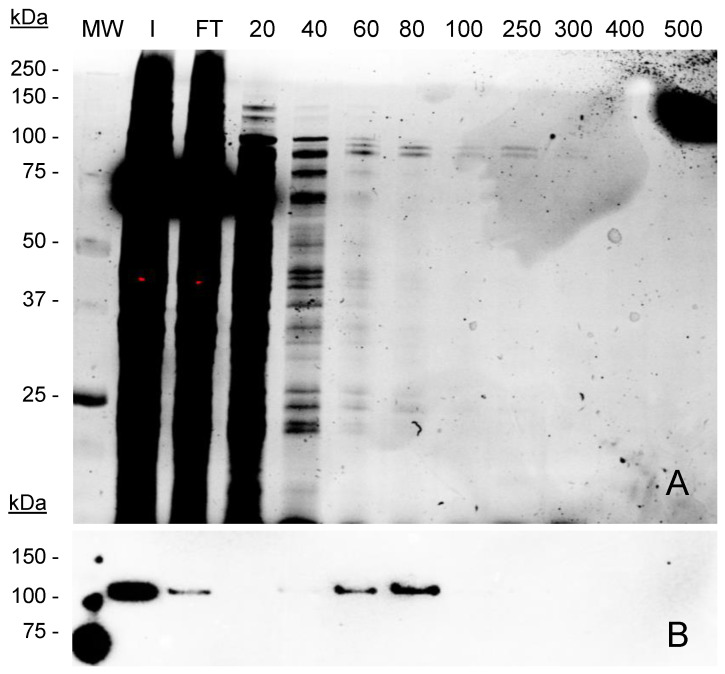
Optimization of conditions for MVP-H6 purification. Purification of MVP-H6 protein with TALON Superflow resin, using different concentrations of imidazol as elution agent. Samples were analyzed by (**A**) SDS-PAGE and TGX visualization, and (**B**) Western blot developed with a monoclonal antibody against His-tag. Legend: initial sample (In), and flowthrough (FT). Numbers 20 to 500 indicate imidazol concentration (mM) used in each step. MW: molecular weight standard.

On the other hand, the used washing conditions (20 mM imidazol) were not able to eliminate all the contaminants attached to the resin in a non-specific manner, since the first elution fraction still contained a significant amount of such contaminants (as seen in Figure 4A, lane E1). Therefore, the next step aimed to determine the optimal washing and elution conditions to reach optimal yield and purity. For that, the experiment was repeated but testing increasing amounts (20 to 500 mM) of imidazole. The results (Figure 5) showed that significant amounts of proteins are initially captured by the resin in a non-specific manner. Such contaminants are eliminated by washing with imidazol at 20 and 40 mM, concentrations that are not able to elute MVP-H6 protein. At higher imidazol concentrations, His-tagged protein is eluted with a significant purity. From these results, washing and elution steps were set up for further experiments at 40 mM and 300 mM imidazol, respectively.

### 2.3. Physico-Chemical Characterization of Recombinant Nanoparticles

**Figure 6:**
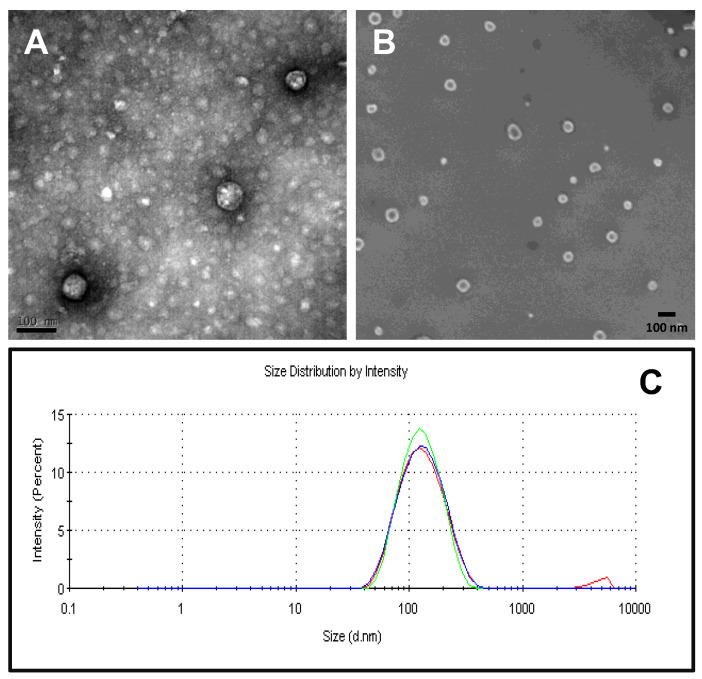
Physico-chemical characterization of purified vault-based nanoparticles. (**A**,**B**) show transmission and scanning electron microscopy images of purified vault-based nanoparticles, respectively. In both cases, bars (in black) correspond to 100 nm. In (**C**), nanoparticles size is estimated (triplicate readings shown) by dynamic light scattering analysis, showing a main peak at approx. 130 nm.

The purified MVP-H6 was visualized by TEM and SEM (Figure 6A,B, respectively) to evaluate if this protein displayed self-assembly capacities, and by DLS (Figure 6C), to estimate the size distribution of the putative resulting NPs.

The obtained electron microscopy images showed the presence of pseudo-spherical NPs, confirming that the MVP-H6 monomer produced in bacterial cells maintains its self-assembling properties and is able to form vault-like NPs. It is noticeable that these NPs display a spherical shape, different from natural or recombinant vaults produced in other expression systems, such as mammalian cells [37]. The NP size was estimated to be around 70–80 nm. In addition, it was possible to distinguish in SEM micrographs smaller aggregates of ~20 nm, which might correspond to another type of nanostructure formed by unspecific self-assembly of the protein. On the other hand, DLS analyses showed a highly monodisperse NP population, as determined by the obtained low polydispersity indexes (0.28 ± 0.007), and with a hydrodynamic diameter of approx. 130 nm. This discrepancy between the estimation of NP size by different techniques was already observed for recombinant vaults produced in a human cell line [37], and it will need further elucidation. However, since NP size according to electron microscopy images (around 70–80 nm) is in agreement with that previously described for native and recombinant vaults, we suggest that it is a more realistic estimation, and it corresponds (as described for other recombinant vaults) to the real NP size.

### 2.4. GFP-INT Protein Expression and Solubility

**Figure 7:**
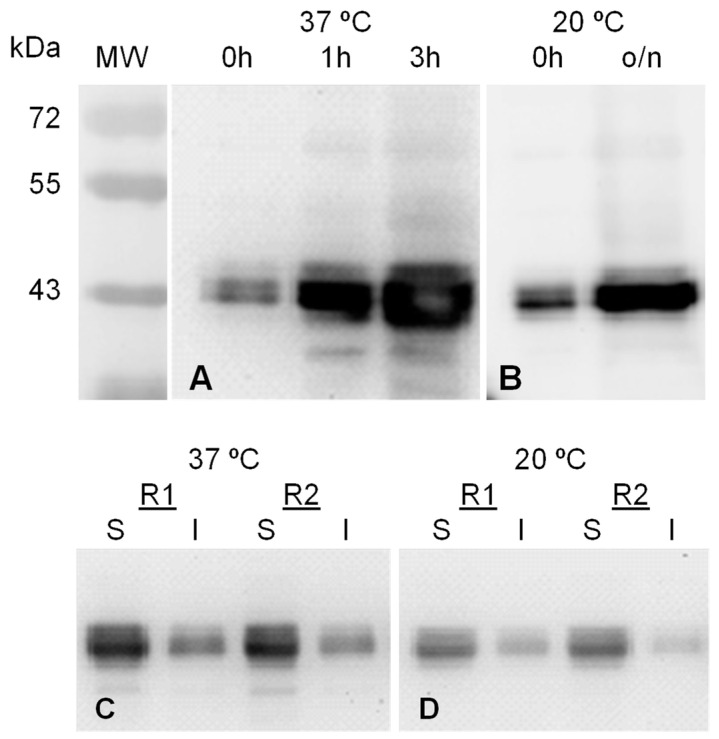
Production (top) and solubility (bottom) of GFP-INT expressed under two different inducing conditions. Samples of total cell pellets of induced BL21(DE3)pLysS/pTriEx1.1-GFP-INT cultures at 37 °C for 3 h and o/n at 20 °C ((**A**,**B**), respectively), and their corresponding soluble (S) and insoluble (I) cell fractions (**C**,**D**) were analyzed by SDS-PAGE and further western-blot developed with an anti-GFP antibody.

GFP-INT expression was induced also by IPTG addition and further incubation either 3 h at 37 °C or o/n at 20 °C. GFP-INT was produced under both conditions, rendering a polypeptide of the expected molecular mass (approx. 44 kDa), which accumulates within the cells after IPTG addition (Figure 7A,B).

To evaluate GFP-INT solubility, induced cells were lysed and soluble and insoluble fractions were analyzed by SDS-PAGE and Western blot. The results (Figure 7C,D) showed that, under both induction conditions, GFP-INT is mainly found in its soluble form. When produced at 37 °C for 3 h or o/n at 20 °C, 71.1 ± 1.6% and 76.9 ± 2.7% of the protein remained within the soluble fraction, respectively. According to these results, and to standardize the production conditions for MVP-H6, induction o/n at 20 °C was chosen to produce the recombinant GFP-INT for further experiments.

### 2.5. Encapsulation and Purification of GFP-INT

**Figure 8:**
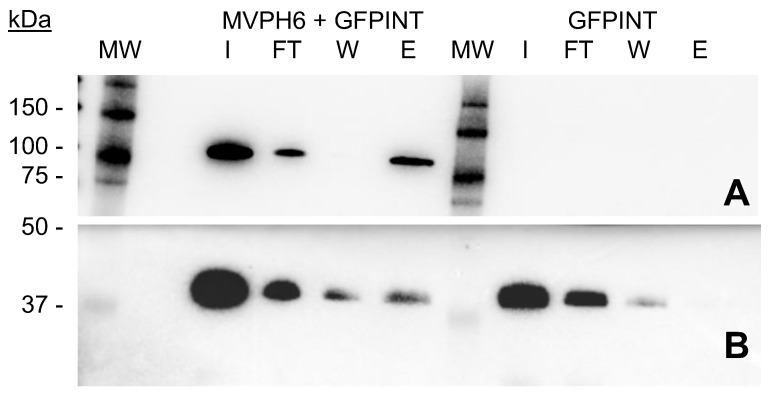
Co-purification of MVP-H6 and GFP-INT proteins. IMAC-based resin was used to purify both MVP-H6 and GFP-INT, as described in Material and Methods. Images correspond to SDS-PAGE and Western blot developed with an anti-His monoclonal antibody (**A**) or a polyclonal antibody anti-GFP (**B**). Legends: initial sample (I), flowthrough (F), wash (W) and eluted (E).

To explore the putative encapsulation of GFP-INT fusion within vault-like NPs, soluble fractions from cell lysates containing either MVP-H6 or GFP-INT were mixed and incubated 1 h on ice. As a control, a mixture containing only GFP-INT with PBS was used. Then, both sample and control were submitted to IMAC-based purification. For that, and to avoid the drawbacks previously detected for MPs, we used TALON resin to purify GFP-INT loaded nanoparticles. The fractions obtained during the process were analyzed by SDS-PAGE and Western blot. The results (Figure 8) confirmed that GFP-INT is not specifically captured when presented alone to the resin. Therefore, the protein is mainly found in the FT and Wash fractions, indicating that, as expected, it cannot directly interact with ions present in the resin. However, when both MVP-H6 and GFP-INT are mixed, a significant amount of GFP-INT is clearly detected in the eluted fraction. In this situation, MVP-H6 is able to interact with GFP-INT, which is further copurified and found in the eluted fraction. Since GFP-INT lacks a His-tag, and therefore, it is not supposed to interact with the resin (as shown by the control), the results strongly suggest that GFP-INT is captured and co-purified by the resin through its interaction with MVP-H6.

**Figure 9:**
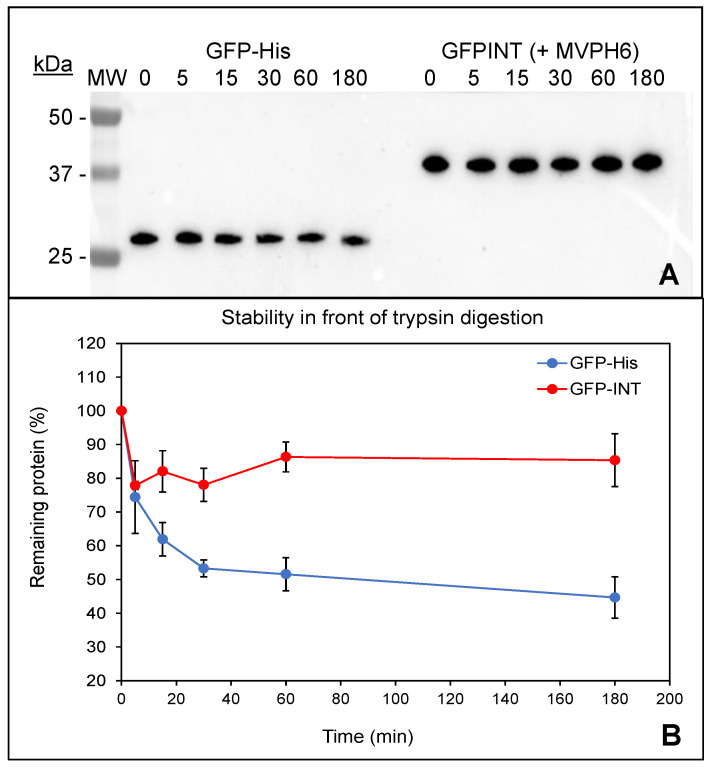
Trypsin digestion of GFP-His and GFP-INT. Samples at the indicated time points (in minutes) were taken and analyzed by SDS-PAGE and Western blot developed with a polyclonal antibody anti-GFP (**A**) detecting both GFP-H6 and GFP-INT. Kinetics of trypsin degradation according to densitometric analysis of Western blots is shown in (**B**). Results shown represent the mean and standard error of the mean (SEM) of two independent samples analyzed by duplicate.

Protection of cargo molecules in front of proteolytic digestion is one of the main advantages provided by encapsulation processes. Therefore, and in order to gain further insight regarding putative encapsulation of GFP-INT protein within vault-like NPs, trypsin digestion was used to explore if vault-like NPs confer any protection to GFP-INT. This would be indicative that GFP-INT is somehow “surrounded” by the nanostructure formed by MVP-H6 protein. As a control of “naked” and therefore unprotected protein, we used a purified His-tagged GFP. Our hypothesis was that GFP-INT protein would be less accessible to trypsin attack due to its interaction (and protection) with MVP-H6, while unprotected GFP-His would be more rapidly degraded. After trypsin treatment of both samples, the results (Figure 9) showed a significant difference in the degradation pattern of GFP-His and GFP-INT proteins. Unprotected GFP-His is steadily degraded by trypsin, with ~45% of protein remaining after 3 h of treatment. On the other hand, under the same conditions, ~15% of GFP-INT seems to be rapidly degraded in the first minutes of treatment, but after that, the remaining 85% of the protein remains intact even after 3 h of treatment. All these results could indicate that even though a small percentage of GFP-INT seems to be exposed and accessible to proteolytic attack, most of the protein is, if not fully encapsulated, at least protected. Such protein remains reluctant to trypsin action, suggesting that interaction with vault-like NP results in a significant degree of protection against proteolytic attacks.

## 3. Discussion

The properties shown by eukaryotic vaults have raised interest in such protein-based NPs as promising DDSs [16]. Recombinant vaults are currently produced in *Sf*9 insect cells [19], one of the few eukaryotic cells lacking endogenous vaults. This complex expression system requires costly and time-consuming processes, including not only the obtention of the recombinant baculovirus but also its further growing, titration and maintenance. In this context, it is surprising that (to our knowledge) the production of vault-like NPs in a prokaryotic expression system has not been previously reported, since these systems are usually faster and cheaper than eukaryotic ones. Despite all the literature published regarding different aspects of vaults, the production of recombinant vaults has been only described in a very few expression systems, all of them eukaryotic. The complexity of vault NPs could be a reason why eukaryotic cell factories have been the first choice. Due to vault NPs’ unique assembly process, a challenge stands out with prokaryotic vault synthesis. Due to differences between polyribosomes topology and structure among eukaryotic and prokaryotic cells, it was unknown whether prokaryotic polyribosome could direct vault self-assembly as has been proposed for eukaryotic cells [29]. This could be another reason why bacterial cells have been neglected for vault production purposes. As a promising alternative, we propose the production of vault-like nanocarriers in a prokaryotic expression system like *E. coli*. In the present work, we have successfully produced a His-tagged version of the MVP protein in *E. coli* cells by standard, well-established procedures based on IPTG-induced gene expression. The MVP-H6 protein was correctly produced and identified by antibodies against both MVP and His-tag domains. Moreover, we confirmed by electron microscopy techniques and DLS that recombinant MVP-H6 retains its self-assembly properties, rendering pseudo-spherical NPs (an unexpected shape not in accordance with the barrel-shape of native and other recombinant vaults). It is well-known that the size of NPs is of paramount importance for the use of these structures as DDS. Particle size strongly affects bioavailability and blood circulation time [38,39]. After systemic administration, particles below 10 nm are rapidly eliminated through extravasation and renal clearance [40], while those bigger than 200 nm are usually sequestered by the spleen, lung and liver [41] and removed by phagocytes [42]. Particles from 70 to 200 nm show the most prolonged circulation times [38]. In this line, vault-like NPs produced in bacterial cells would represent an excellent DDS candidate in terms of size parameter, since dimensions rendered by TEM (between 70 and 80 nm) fall within the optimal range described to interact with and internalize within target cells.

Due to the used expression system (lacking endogenous MVP protein), we can assume that a single NP is basically composed by the recombinant MVP-H6. Thus, the different shape observed here is an intriguing fact that needs further study to obtain deeper insights. Moreover, the presence of smaller NPs (approx. 20 nm in size) visible in SEM images suggests that the folding and self-assembling of the MVP-H6 monomer into vault-like NPs clearly differs when directed by a prokaryotic synthetic machinery. As previously described [29], the self-assembly of MVP into vaults is a complex, well-orchestrated process requiring dozens of well-organized ribosomes (“polyribosome”) working simultaneously on a single MVP mRNA. Differences between prokaryotic and eukaryotic ribosomes could explain, at least partially, the appearance of these “secondary” self-organization pathways rendering smaller nanostructures (not detected when expressing human vaults in different eukaryotic cell factories), and the difference in shape with native vaults. Whether the different polyribosome topologies found in prokaryotes and eukaryotes are related and responsible of the particular shape and sizes seen in vault-like NPs produced in a bacterial cell factory awaits further studies and a deep structural and mechanical characterization of self-assembling process in prokaryotic cell factories.

However, these structural differences did not seem to hamper the ability of our recombinant vault-like NPs to capture the INT-tagged cargo protein. The encapsulation of many INT-tagged proteins within recombinant vaults has been reported [43]. Such encapsulation was achieved by the co-infection of *Sf*9 cells with recombinant baculoviruses encoding either the MVP or the INT-tagged protein, or by simply mixing both components and incubating on ice. Again, this approach needs the previous construction of the respective recombinant baculoviruses. To overcome this drawback, we propose the straightforward mixing of soluble fractions from bacterial cells producing MVP-H6 and GFP-INT, thus taking advantage of the “breathing” event described for vaults [21]. This phenomenon consists of the opening/closing of the two halves forming the mature vault structure, allowing the spontaneous entrapment of INT-containing proteins within the vault lumen. Thus, when mixing both MVP-H6 and GFP-INT proteins, the INT domain acts as a targeting peptide, directing the close interaction of both proteins, as later demonstrated by the co-purification of the loaded NP through the His-tag fused to MVP protein. Moreover, the interaction of the cargo protein GFP-INT with vault-like NPs seems to confer a significant degree of protection against proteolytic attack. Due to the unexpected final architecture obtained when MVP-H6 protein is expressed in *E. coli* cells, a deeper structural study is needed to elucidate in the future the fine architecture of vault-like NPs obtained in prokaryotic cell factories. However, all the preliminary results presented here strongly suggest that such prokaryotic vault-like NPs are able to encapsulate cargo proteins by the well-known interaction between INT domain and MVP protein. These results also encourage the use of these nanovehicles produced in a cheap and easy expression system like *E. coli*.

Despite the increasing interest raised by vaults, their purification is still complex and labor intensive. Ultracentrifugation in sucrose or cesium chloride gradients is considered appropriate to purify protein-based NPs such as virus-like particles (VLPs) [44,45], but it is labor-intensive, time-consuming and scale-restricted [46]. Therefore, the development of faster and easier down-stream procedures for recombinant vaults is needed. In this line, new approaches have been described in the literature, such as the use of ion exchange columns [47], ion exchange followed by gel filtration [30] or dialysis combined with size exclusion chromatography [48]. However, vault purification is still performed by the original protocol based on several ultracentrifugation steps. In this line, a significant result of our study is the possibility of purifying recombinant vaults by means of well-known affinity chromatography strategies. Magnetic purification is a fast, gentle, scalable and easily automated procedure, and it has been applied to fields such as wastewater treatment, molecular biology, cell sorting or clinical diagnostics [49,50]. Following this rationale, we used commercial MPs to purify recombinant vaults. This approach allowed us to obtain vault-based NPs with a significant degree of purity by a fast and easy protocol. A common issue for MPs that would need further study and optimization is the low capture efficiency (“binding capacity”) of these materials compared to other supports. In addition, another issue needing further study is the optimization of the elution conditions, since NPs remains strongly attached to MPs, diminishing the overall efficiency of this down-stream process. This fact surely hampers the replacement of current down-stream procedures by magnetic-based ones. However, an IMAC-based resin used in solution allowed the easy and fast purification of vault-like NPs by well-known protocols, avoiding drawbacks of current purification methods and increasing the toolbox to obtain pure vault-like NPs for diverse applications.

In conclusion, we describe in this work the production in a prokaryotic expression system (*E. coli* cells) of vault-like NPs and the spontaneous interaction and encapsulation (according to their co-purification profile) of an INT-tagged GFP within such NPs. In our work, we have demonstrated that, irrespective of the differences between prokaryotic and eukaryotic cells and their protein synthesis machinery, bacterial cells are able to synthesize MVP protein and, most importantly, that this protein is able to self-assemble into NPs with appealing applications in biotechnology and biomedicine.

The main intrinsic advantages of prokaryotic cell factories to obtain such vault-based NPs can be of great help not only in the design and obtention of new drug delivery systems, but also in the understanding of differences between eukaryotic and prokaryotic protein synthesis machineries when translating a polypeptide with self-assembling capabilities. Altogether, these results prompt us to propose *E. coli* as a new cell factory in a promising strategy to produce (in very short times) significant amounts of recombinant vaults and their variants. Further work is still needed to elucidate the structural characteristics of vault-based NPs produced in a prokaryotic cell factory, but the faster, cost-effective protocols presented here will surely boost the study of improved DDS based on recombinant vault-based nanovehicles. The combination of a well-known prokaryotic expression system together with robust and versatile IMAC-based chromatographic methods will surely speed basic research and the putative use of vault-based nanovehicles.

## 4. Materials and Methods

### 4.1. Synthesis and Cloning of Recombinant Genes

Details of synthetic genes and recombinant plasmids used in this work have been previously described [37]. Briefly, the sequence of the human major vault protein (MVP, UniProt Q14764; MVP_HUMAN) was modified by adding, at its 3′ end, extra triplets encoding a histidine tag (6xHis). To explore protein encapsulation within vault-like NPs, an “enhanced green fluorescence protein” (eGFP) was used. The designed recombinant gene codifies for the eGFP gene fused to the INT domain DNA sequence (amino acids 1562–1724 of poly-(ADP-ribose) polymerase 4, PARP4 protein, Q9UKK3, PARP4_HUMAN). This INT domain promotes the interaction of PARP4 with the MVP monomer. The resulting plasmids, named pTriEx1.1-MVP-H6 and pTriEx1.1-GFP-INT, express, respectively, the recombinant proteins MVP-H6 (899 aminoacids, molecular weight of ~100 kDa) and GFP-INT (401 amino acids, molecular weight of 45.2 kDa). Both MVP-H6 and GFP-INT genes were synthesized and cloned by GeneArt^®^ (a company from Life Technologies, Regensburg, Germany) in the pTriEx1.1-Hygro vector (6951 bp, cat. n° 70928-3, Novagen, a company from Merck, Burlington, VT, USA).

### 4.2. Escherichia coli Strains and Expression Plasmids

The *E. coli* DH5α strain was used to maintain the expression plasmids used in this work. To express the different recombinant proteins, the *E. coli* strain BL21(DE3)pLysS was used. In this strain, the expression of the T7 lysozyme gene encoded in pLysS provides a tight control of recombinant gene expression.

### 4.3. Recombinant Protein Expression

Recombinant MVP-H6 and GFP-INT proteins were produced in the BL21(DE3)pLysS strain by induction at mid-log growth phase by isopropyl β-D-1-thiogalactopyranoside (IPTG) addition (0.1, 0.5 or 1 mM final concentration). After induction, cells were incubated at two conditions (overnight (o/n) at 20 °C, or 3 h at 37 °C) on incubator shakers set at 250 rpm. To check protein expression, 1 mL samples from induced cultures were taken at different times (at 0 h and 3 h from cultures incubated at 37 °C, or at 0 h and o/n from cultures incubated at 20 °C). These samples were centrifuged (13,400 rpm, 10 min), and after that, supernatants were discarded. Cell pellets were resuspended in Laemmli buffer to analyze them by sodium dodecyl sulphate polyacrylamide gel electrophoresis (SDS-PAGE).

### 4.4. Bacterial Cell Fractionation

After the induction of protein expression, bacterial cells were harvested by centrifugation (13,400 rpm, 10 min). Cell pellets were resuspended with phosphate-buffered saline (PBS) supplemented with the protease inhibitor Complete EDTA free (ref. 11873580001, Roche Life Sciences, Penzberg, Germany) and stored at −20 °C until fractioned by sonication. To estimate the percentage of soluble MVP-H6 and GFP-INT proteins, cell pellets in PBS were disrupted by sonication (7 cycles, amplitude 10%, pulse ON/OFF: 0.5 s). Then, sonicated lysates were centrifuged (13,400 rpm, 10 min), and supernatant containing soluble proteins was separated. The pellets containing the insoluble fractions were resuspended with PBS in the same volume as soluble fractions.

### 4.5. SDS-PAGE and Western Blot Analyses

To detect and quantify the recombinant proteins, SDS-PAGE was performed by using TGX Stain-Free™ FastCast™ acrylamide 12% (ref. 161-0185, Bio-Rad, Hercules, CA, USA), and further visualization of proteins with a ChemiDoc™ Touch Imaging System (Bio-Rad). For Western blot analyses, a rabbit polyclonal antibody anti-MVP (ref. ab90009, Abcam, Cambridge, UK) or anti-His mouse monoclonal antibodies (ref. A00186-100, GenScript, Piscataway, NJ, USA, or ref. 631212, Clontech, Mountain View, CA, USA) were used as primary antibodies to detect MVP-H6 protein. GFP-INT fusion protein was detected with a rabbit polyclonal antibody anti-GFP (ref. sc-8334, Santa Cruz Biotechnology, Dallas, TX, USA). MVP-H6 amounts were densitometrically estimated by comparison, after SDS-PAGE and western-blot analyses, with known amounts of a commercial MVP (ref. ab153030, Abcam, Cambridge, UK). Samples and standards to be quantitatively compared were run in the same gel and processed as a set. Densitometric analyses of the immunoreactive bands were performed with Image Lab™ software (version 6.0.1., Bio-Rad, Hercules, CA, USA).

### 4.6. Protein Purification

In all cases, samples used in the purification experiments were soluble fractions obtained from induced (o/n at 20 °C) cultures. The purification of MVP-H6 was carried out using two different cobalt-based immobilized metal affinity chromatography (IMAC) platforms, which typically provide higher purity than nickel-based resins. The first one was the superparamagnetic particles (MPs) “Dynabeads His-tag isolation and Pull-down” (ref. 10103D, Invitrogen, Waltham, MA, USA). MPs were handled as indicated by the vendor, using a magnetic separator (ref. S1506S, New England Biolabs, Ipswich, MA, USA). The second purification platform tested was the Talon^®^ Superflow™ resin (ref. 28-9575-02, Cytiva, Marlborough, MA, USA). Briefly, purification protocols were as follows. Dynabeads MPs were washed twice with PBS and then mixed with bacterial soluble fractions containing the recombinant MVP-H6. After incubating on a roller, MPs were magnetically immobilized, and flowthrough fraction was recovered. Then, MPs were washed with 5 mL of 20 mM imidazole in PBS and magnetically immobilized, and the wash fraction was collected. Finally, captured MVP-H6 was eluted from MPs with 2 mL of elution buffer (250 mM imidazole in PBS). To check elution efficiency, a second elution step (with 2 mL of 2 M imidazole) was performed. Finally, MPs were washed with PBS, and a sample was taken for further testing. All steps were performed by incubating the MPs on a roller for 15 min at room temperature. In the case of TALON resin, typically, 2 mL of slurry was packed into an empty column, washed with 5 mL of distilled H_2_O and equilibrated with 5 mL of PBS. Then, the sample was mixed with the resin and incubated for 30 min, carefully resuspending the mixture several times during the incubation period to obtain a good interaction between resin and sample. The flowthrough fraction was recovered, and the resin was washed twice with 2.5 mL of wash buffer (20 mM imidazole). Captured protein was eluted by incubating (5 min) with 2 mL of elution Buffer (250 mM Imidazole). To check the elution efficiency, a second elution step (with 2 mL of 2 M imidazole) was performed. Finally, the resin was washed with 2 mL of PBS, and a sample was taken for further testing. For a further optimization of washing and elution conditions, the experiment was repeated to test increasing imidazole concentrations (from 20 to 500 mM) in PBS.

### 4.7. Physico-Chemical Characterization of Recombinant Vault-like Nanoparticles

The samples containing MVP-H6 were dialyzed in dialysis cassettes (Slide-A-Lyzer 3.5K Dialysis Cassettes, Thermo Fisher Scientific, Waltham, MA, USA) against buffer A (Tris-HCl 50 mM pH 8, NaCl 75 mM, MgCl_2_ 0.75 mM). Dialyzed samples were analyzed using transmission electron microscopy (TEM). For that, they were adsorbed (5 µL) onto Cu-C grids (2 min, room temperature), dried and negatively stained (2 min, room temperature) with 5 µL of 2% uranyl acetate. Finally, the samples were air-dried prior to viewing in a JEOL JEM 1400 microscope. For the analysis by scanning electron microscopy (SEM), 10 μL protein samples were used. Samples were placed into a silicon substrate, air-dried and observed without coating. Images were acquired in a Zeiss Merlin operating at 1 kV with a high-resolution in-lens secondary electron detector. The size distribution of vault-based NPs was estimated by dynamic light scattering (DLS) at 633 nm wavelength, combined with non-invasive backscatter technology (NIBS) in a Zetasizer Nano ZS (Malvern Instruments Limited, Malvern, Worcestershire, UK). Before analysis, samples were filtered to avoid putative interferences due to remaining traces of MPs used for purification. The mean value of three measurements was taken as the hydrodynamic NP diameter.

### 4.8. GFP-INT Protein Encapsulation

The putative spontaneous encapsulation of GFP-INT protein inside MVP-H6-based nanocapsules was checked by mixing (60 min on ice) soluble fractions obtained from induced cultures (o/n, 20 °C) expressing both proteins. As a control, a soluble fraction containing only GFP-INT was mixed with PBS and further processed as the mixture containing both proteins. After incubation, MVP-H6-based NPs were purified as previously described, and samples obtained during the process were submitted to SDS-PAGE and Western blot. As a second approach to check interaction between vault-like NPs and GFP-INT, a proteolytic digestion with trypsin (ref. T7409, Sigma-Aldrich, Burlington, VT, USA) was performed over co-purified vault-like NPs and GFP-INT, using as a control a purified His-tagged GFP (namely GFP-His). For that, both samples (at 0.02 µM) were mixed with trypsin (final concentration of 0.01 µM) and incubated at 37 °C for 3 h. At different time points, the samples were taken and immediately mixed with denaturing loading buffer to stop proteolytic reaction. Finally, all the samples were analyzed by SDS-PAGE and Western blot developed with a rabbit polyclonal antibody anti-GFP and anti-His mouse monoclonal antibodies. The remaining proteins were densitometrically estimated by comparison with protein at time zero, taken as 100%.

## Data Availability

All data generated or analyzed during this study are included in this published article.
